# Potential Applications of Mitochondrial Therapy with a Focus on Parkinson’s Disease and Mitochondrial Transplantation

**DOI:** 10.34172/apb.2024.019

**Published:** 2023-10-14

**Authors:** Pranay Wal, Ankita Wal, Himangi Vig, Danish Mahmood, Mohd Masih Uzzaman Khan

**Affiliations:** ^1^Pharmacy Department, PSIT- Pranveer Singh Institute of Technology, (PHARMACY) Kanpur-Agra-Delhi National Highway (NH-2), Bhauti-Kanpur-209305.; ^2^Department of Pharmacology and Toxicology, Unaizah College of Pharmacy, Unaizah 51911, Saudi Arabia.; ^3^Department of Pharmaceutical Chemistry and Pharmacognosy, Unaizah College of Pharmacy, Unaizah 51911, Saudi Arabia.

**Keywords:** Mitochondrial dynamics, Mitochondrial therapeutics, Mitochondrial transplantation, Neurodegeneration, Parkinson’s disease

## Abstract

**Purpose:**

Both aging and neurodegenerative illnesses are thought to be influenced by mitochondrial malfunction and free radical formation. Deformities of the energy metabolism, mitochondrial genome polymorphisms, nuclear DNA genetic abnormalities associated with mitochondria, modifications of mitochondrial fusion or fission, variations in shape and size, variations in transit, modified mobility of mitochondria, transcription defects, and the emergence of misfolded proteins associated with mitochondria are all linked to Parkinson’s disease.

**Methods:**

This review is a condensed compilation of data from research that has been published between the years of 2014 and 2022, using search engines like Google Scholar, PubMed, and Scopus.

**Results:**

Mitochondrial transplantation is a one-of-a-kind treatment for mitochondrial diseases and deficits in mitochondrial biogenesis. The replacement of malfunctioning mitochondria with transplanted viable mitochondria using innovative methodologies has shown promising outcomes as a cure for Parkinson’s, involving tissue sparing coupled with enhanced energy generation and lower oxidative damage. Numerous mitochondria-targeted therapies, including mitochondrial gene therapy, redox therapy, and others, have been investigated for their effectiveness and potency.

**Conclusion:**

The development of innovative therapeutics for mitochondria-directed treatments in Parkinson’s disease may be aided by optimizing mitochondrial dynamics. Many neurological diseases have been studied in animal and cellular models, and it has been found that mitochondrial maintenance can slow the death of neuronal cells. It has been hypothesized that drug therapies for neurodegenerative diseases that focus on mitochondrial dysfunction will help to delay the onset of neuronal dysfunction.

## Introduction

 In most eukaryotic organisms, the mitochondria are a double membraned organ in the body. Mitochondria come in a variety of sizes, from 0.75 to 3 µm in diameter, but their extensions and structures differ. Many mitochondria construct cable-like networks and use the oxidative phosphorylation mechanism to fulfill specific cell requirements. Mitochondria host a variety of biosynthetic mechanisms, assist in energy generation, and manage apoptosis. It is involved in amino acid biosynthesis, increased lipid peroxidation, steroid biosynthesis, intermediary metabolic functions, calcium homeostasis, and oxidative stress scavenger.^1^

 It performs several functions, many of which are vitally pertinent for neuronal survival. Mitochondria are vital organelles in many types of cells; however, they serve a special role in the central nervous system. Energy generation, Ca^2+^ modulation, the potential of plasma membrane regulation, axons and dendrite transit, and the discharge and reuptake of neurotransmitters at the synaptic junction are all dependent on mitochondrial function. An expanding number of studies have found that mitochondrial defects contribute to aging, age-related neurological diseases, and a variety of other mitochondrial related disorders.^2^ Monoenzymatic complex malfunction, oxygen radical’ formation, mitochondrial permeability transition pore formation, increased cell cycle arrest, and mitochondrial structural abnormalities are regarded as significant for the commencement and development of neurological diseases.^3^

 The changing nature of mitochondria, which is characterized by tightly controlled fission and fusion, affects mitochondrial integrity and performance. Mitochondrial malfunction is a symptom of Parkinson’s disease (PD). Mitochondria can alter neuronal function by regulating ion homeostasis (particularly Ca^2+^ ), synaptic performance, oxidant generation, cell communication, and viability, in addition to ATP production. Aging and oxidative injury increase when mitochondrial point mutations accumulate. This is likely why neurodegenerative disorders like Parkinson’s are age-dependent.^4^ Variations in specific proteins that alter mitochondria cause a variety of neurodegenerative disorders. PD is the result of variations in the nuclear-encoded proteins PINK1 (PTEN-induced putative kinase 1), DJ-1, and Parkin, which damage mitochondria. Such observations add to the new source of proof that medicines that increase mitochondrial biogenesis, aim for the mitochondrial permeability transition, or display antioxidant capability may be effective in the treatment of neurodegenerative disorders. Researchers are focusing their investigation on creating therapeutics, such as compounds that approach and shield mitochondria and neurons from the damage of aging and mutated proteins, as a result of the analysis that is explaining the function of mitochondria in disease genesis and progression. We are convinced that mitochondrial grafting may be a viable treatment option for discovering a conceivably useful treatment for a wide range of mitochondrial illnesses. There are several obstacles to overcome before disease management via mitochondrial transplants can be effectively employed in humans. Mitochondrial transfer procedures have been researched in many ways to enhance mitochondrial uptake and transplanting efficiency, including direct microinjection, cell-mediated distribution via tunneling nanotubes, and systemic administration. These administration strategies, however, have been known to interfere with transplant viability.^5^

 Trials in animal and cellular models of many neurological illnesses reveal that mitochondrial maintenance can help slow neuronal cell death. It has been suggested that pharmaceutical techniques to alleviate neurodegenerative disorders that target mitochondrial dysfunction will assist in preventing the development of neuronal dysfunction. This article analyzes mitochondrial defects and pathologies in neurodegeneration, as well as some emerging mitochondrial therapeutic applications in neurodegenerative disorders therapy.

###  Anatomy and biogenesis of healthy mitochondria

**Figure 1:**
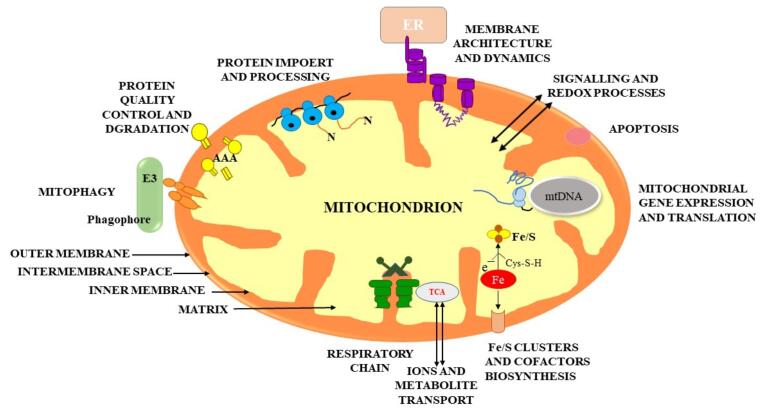
Shows the basic structure of the mitochondria and also highlights the physiological mechanism taking place inside the mitochondria namely, metabolism with ATP generation and respiration; metabolism of lipids, amino acid, and nucleotide; biogenesis of (Fe-S) groups and cofactors; mitochondrial genomic expression; mitophagy and cell death; signalling and reaction kinetics mechanisms; membrane construction and complexities; and import and handling of precursor proteins

 The extracellular and intracellular mitochondrial membranes are the two lipid membranes that surround mitochondria. The external membrane is permeable, enabling low-molecular-weight particles to diffuse between the cytosol and the mitochondrial intermembrane region. The internal membrane serves as a highly effective ion transport barrier, harbors the mitochondrial respiratory cycle or electron transport chain, and protects the mitochondrial structure. Mitochondrial proteins, DNA and RNA polymerases, biochemical enzymes, associated proteins, and mtDNA regulatory changes like mitochondrial transcription factor A are all encoded by nuclear genes.^6^ The mechanics of the whole bilayer (referred to as “fluidity”) control a multiplicity of important cell mechanisms, like the operations of the of membrane-related enzymes, transporters, and ion channels^7^ (Figure 1).

 The principal objective of mitochondria is to generate energy from adenosine triphosphate by oxidative phosphorylation, which involves electrons being moved along the electron transport chain (ETC) while a proton gradient is generated. The adenosine triphosphate (ATP) synthase enzyme is driven by this gradient.^8^ The Na + /K + and Ca2 + ATPases, which regulate ion gradients, require neural ATP to operate. Conversely, mitochondria perform a key role in Ca^2+^ buffering by utilizing ion exchangers to sequester Ca^2+^.^9^ Mitochondrial actions are particularly critical for neural transmission, synapse generation, and maintenance.^10^

 Mitochondrial biosynthesis is modulated following growth, cell proliferation, and alterations in oxidizing stimuli and hormones to adjust the mitochondrial composition to a cell’s energy requirements. The integrated synthesis of nuclear and mitochondrial DNA is essential for mitochondrial biogenesis. The biosynthesis of mitochondria is regulated by respiratory factors 1 and 2 (NRF1 and NRF2).^11^ Sirtuins, AMP-activated protein kinase (AMPK), and the peroxisome proliferator-activated receptors (PPAR) coactivator 1 group of transcription coactivators are also engaged in gene expression regulation throughout mitochondrial biogenesis.^12^

###  Fission and Fusion dynamics in the mitochondria

**Figure 2:**
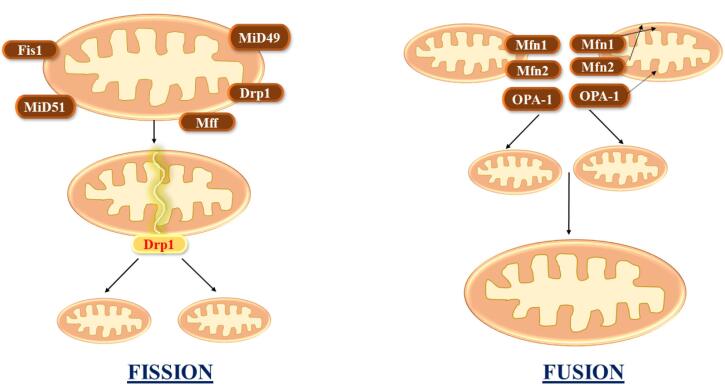
Mitochondrial fusion combines multiple mitochondria, whereas fission divides it into two. The MFN1 and MFN2 regulate fusion on the OMM. Fission starts whenever the endoplasmic reticulum is attracted to the constriction point, which is identified by mtDNA. Following that, FIS1, MFF, MiD49, and MiD51 recruit DRP1 to the mitochondrial surface, assisting in ER-mediated constriction

 Mitochondria are extremely dynamic structures in the cell that proliferate and merge with one another as the physiological state requires. Mitochondrial fusion–fission processes are vital in a variety of cell activities (Figure 2).^13^ These processes seem to be altered in numerous neurodegenerative illnesses, as they regulate mitochondrial architecture, which impacts several critical mitochondrial features such as mitochondrial energy metabolism and quality control. In addition, these processes have been shown to be critical for cell processes.^14^

 Mitochondrial fusion, in particular, allows the interchange of mitochondrial components such as lipid membranes, mtDNA, and oxidative phosphorylation complexes, lowering the fraction of mitochondria with defective components. Defective or impaired fission or fusion has a significant effect on mitochondrial biogenesis, leading to increased free radical formation, distorted mitochondrial enzymatic reactions, poor calcium homeostasis, low ATP synthesis, and general reductions in energy management.^15,16^

 The fusing of biomembranes has several common motifs, according to findings on virus-mediated and SNARE-mediated membrane fusion. The creation of specific protein aggregates in trans among the donors and recipients’ membranes. ensures the specialization of membrane fusion^17^ The creation of extremely reliable helical bundles drives membrane merging. The creation of the trans-SNARE combination result in a very strong four-helix bundle, which effectively drives the donor and acceptor membrane bilayers into close contact during vesicle fusion. Mitochondrial fusion appears to have these characteristics, and mitofusins likely play a role in both. Mfn1 and Mfn2 are proteins that are essential for mitochondrial fusion and are found in the exterior mitochondrial wall, where they might trigger the association of mitochondria with one another. Moreover, during the transmembrane fusion process, mitofusins must be present in nearby mitochondria.^18^ As a result, mitofusins are likely to build aggregates in trans, that is, among nearby mitochondria. According to biological pathways, Mfn1’s C-terminal domain, which contains the hydrophobic heptad repeat sequence (HR2), can oligomerize with itself and with Mfn2’s homologous HR2 sequence. Mfn1 HR2 generates a dimeric antiparallel coiled helix with transmembrane domains on both ends and a diameter of 95 Å. Such a configuration in a trans group would result in tight adhesion of mitochondria but leave a substantial gap. The Mfn1 structure quite probably represents an Mfn1 configuration necessary for mitochondrial attachment, but not actual fusion.^19^ Fusion would necessitate a structural shift that allows mitochondrial membranes to be closer together.

**Figure 3:**
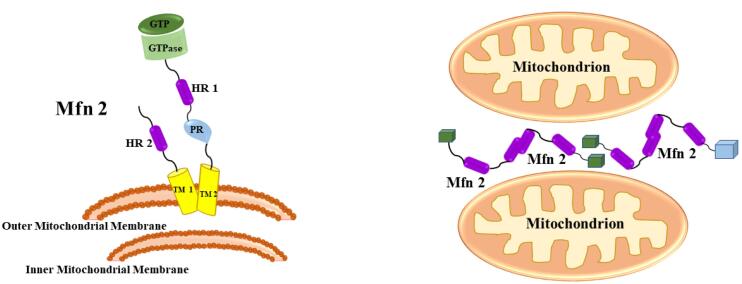
Mitofusins: Mfn1 and Mfn2 presence and role during mitochondrial fusion. A GTPase, a spiral coil (HR1), and a proline-rich (PR) regions are accessible in the cytosol, a transmembrane motif crosses the outer membrane, and a secondary spiral (HR) C-terminal extension makes its way into the intermembrane space

 Mitochondrial fusion necessitates the blending of the composition of the matrix, implying that the exterior and interior layers should fuse in unison. Given the complicated shape of the interior membrane, the synchronization of exterior and interior membrane fusion is a significant accomplishment.^20^ Mfn1 and Mfn2 are shown to be associated with mitochondrial fusion identically (Figure 3). Mfn1 takes a somewhat more active role in themechanism.

 Mitochondrial fission 1 protein (Fis1) and dynamin-related protein 1 (Drp1) are both known to be essential for mitochondrial fission.^21^ Drp1 migration to mitochondria is a vital step in commencing the fission procedure because only a portion of these areas become real fission centers.^22^ Fis1, which is consistently dispersed to the outer mitochondrial membrane, is essential for Dnm1 (also known as Drp1) engagement. Fis1 has a concave area in its cytosolic region that serves as an interaction region. By attaching to Fis1 and Dnm1, Mdv1 and Caf4 serve as molecular adaptors. Fis1 must first be engaged before it can recruit Dnm1, and this engagement is irregular. Drp1/Dnm1 mediates fission once recruited to mitochondria, but the exact mechanism is unknown.^23^ Only a minor portion of Dnm1 punctate proceeds to real fission, and advancement is apparently affected by mechanisms that affect Dnm1 assembly and activation. Since Dnm1 migration to mitochondria in the absence of Mdv1is unable to induce fission, Mdv1 is most likely one of these factors.^24^

## Mitochondrial abnormalities involved with neurodegenerative illness

 Most neurodegenerative illnesses, such as PD, are linked with mitochondrial dysfunction. Numerous findings demonstrate that mitochondria play a major role in neurological disorders, involving (1) faulty mitochondrial enzymatic activity,^25^ (2) mitochondrial DNA errors,^26^ (3) mitochondrial malfunction and oxidative load, and^27^ (4) mitochondrial structural reforms.^28^ Researchers discovered that mitochondrial failure was not seen in PD peripheral cells missing mtDNA, implying that mitochondria are essential for mitochondrial biogenesis as the illness progresses in PD.

###  Most prominent neurodegenerative illness due to dysfunctional mitochondria: 

####  Parkinson’s disease

 PD is the most ubiquitous neurological disorder involving, dyskinesia, hypokinesia, stiffness, trembling, and loss of balance, along with non-motor complaints such as autonomic failure, insomnia, anxiety, and cognition. The decline of dopaminergic neuronal cells in the substantia nigra pars compacta (SNpc) is a pathogenic feature of PD, and the appearance of cytosolic and neuritic aggregates is known as Lewy bodies and Lewy neurites. Mitochondria are constantly carried across axons and dendrites in neuronal cells, allowing them to be recruited to crucial subcellular compartments far enough from the nerve cell.^29^ In patients with PD, abnormal mitochondrial morphologies and functionalities are widely acknowledged pathophysiological processes, demonstrating that mitochondrial impairment and oxidative stress play a significant role in PD pathogenesis.^30^

 Energy metabolism deformities, mitochondrial genome polymorphisms, nuclear DNA genetic abnormalities related to mitochondria, modifications in mitochondrial fusion or fission, variations in shape and size, variations in transit, modified mobility of mitochondria, transcription defects, and the appearance of malformed proteins linked with mitochondria are all connected to PD.^31^

####  Parkinson’s disorder pathogenesis and mitochondrial malfunction

*Complex I malfunctions in mitochondria: *In PD, genetic polymorphisms attributed to the mitochondria, as well as environmental toxins such as rotenone and MPTP, cause breakdowns in the electron transport system, resulting in higher oxidative stress, cellular Ca^2+^ buildup, glutamate excitotoxicity, and a decline in producing energy, all of which contribute to neuronal injuries and loss. Because a minor amount of superoxide anion is formed during the reduction of molecular oxygen to water, the ETC is the main cause of ROS. Through oxidative phosphorylation, the ETC generates ATP.^32^ Various blockers of complex I that cause dopaminergic neurotoxicities, such as rotenone, trichloroethylene, and fenpyroximate, show that mitochondrial impairment plays a significant role in PD. Such contaminants lead to failures in the operation of the mitochondrial ETC, minimize mitochondrial mobility,^33^ enhance the mitochondrial permeation transition, boost the production of ROS, and increase the expression of nitric oxide (NO) synthase in the mitochondria.^34^

**Figure 4:**
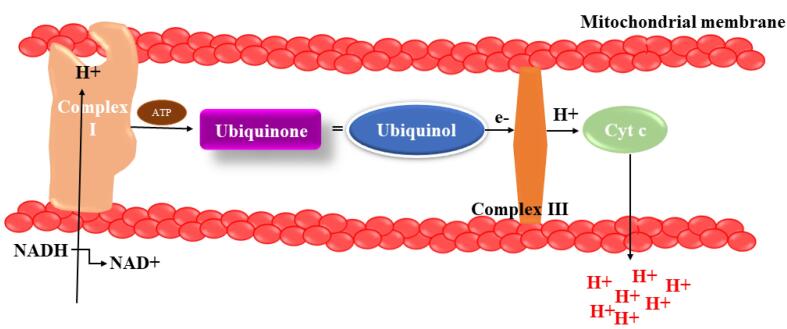
Complex I malfunction in mitochondrion. By donating electrons to Complex III, Complex II connects the TCA pathway to the electron transport system. Complex III serves to the proton gradient by reducing cytochrome c levels through ubiquinone oxidation and causing protons to exit the mitochondrial intermembranous area

 Complex III helps the proton gradient by reducing cytochrome c by ubiquinone oxidation and pushing protons out of the mitochondrial intermembrane space (Figure 4). When electron transport is reduced, molecular oxygen can acquire electrons from Complex III, which is involved in the making of superoxide anion. Nonetheless, it appears that mitochondrial function is disturbed in PD at several levels, including organelle biosynthesis, mitochondrial fusion/fission, and mitophagy.^35^

*Oxidative disbalance in mitochondria*: Oxidative stress happens whenever there’s a disproportion between the number of ROS created and the cellular program’s capability to eliminate specific reactive species, ending in a hazardous state that promotes cell destruction. A growing body of evidence suggests that oxidative stress and mitochondrial malfunction participate in

 the conditions that led to the deterioration of these dopamine neurons.^36^ The stimulation of glial cells is the dominant contributor to oxidative stress, which is emphasized as an essential component in the development of PD.^37^ The generation of reactive oxygen destroys the substantia nigra during the progression of PD causing DNA and protein oxidation, and lipid peroxidation.^38^ Alongside ROS, there are proofs that reactive nitrogen species play a part in the induction of nitrosative stress.^39^ When superoxide interacts fast with nitric oxide (NO), a large amount of peroxynitrite is generated.

**Figure 5:**
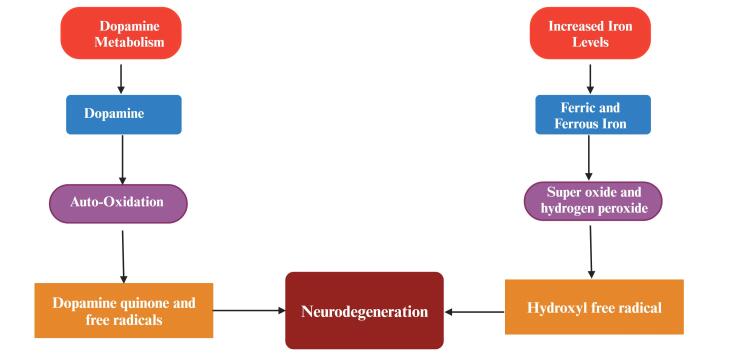
Various pathways responsible for reactive oxygen species generation and neurodegeneration in Parkinson’s disease

 Peroxynitrite, which is a much more aggressive oxidative agent and a more potent oxidizing agent than NO, can cause DNA cleavage and lipid peroxidation.^40^ Peroxynitrite also inhibits dopamine biosynthesis, irrespective of dopamine oxidation or the death of cells. NO suppresses many enzymes, particularly complexes I and IV of the mitochondrial ETC, ending in the generation of ROS (Figure 5).^41^

*Genetic mutations in mitochondria:* Mitochondrial impairment is characterized by mutations in specific gene variants associated with familial versions of the disease. To date, quite a few genes have been detected as hereditary causes of hereditary PD (dominantly inherited SNCA and LRRK2 alterations, as well as recessive Parkin, PINK1, and ATP13A2 polymorphisms). New PD genes were found, including VPS35 and CHCHD2, which emphasise the significance of mitochondrial function in the disease pathogenesis.^42^

 Various other factors related to mitochondrial malfunctioning responsible for PD are mentioned in Table 1.

**Table 1:** summarises the several aspects involving mitochondrial damage that induce Parkinson's disease and their mechanisms

**Factors involved**	**Mechanisms**	**References**
Environmental toxins	1-Methyl 4-phenyl 1,2,3,6-tetrahydropyridine (MPTP) hinders complex-I, reduces ATP generation enhances ROS, hinders complexes III and IV of mitochondria, reduces mitochondrial gene expression, changes metabolic enzymes, oxidative phosphorylation-related proteins, internal and external mitochondrial proteins, changes proteins linked to mitochondrial. Rotenone decreases the action of complex I. Paraquat interacts with free radicals and molecular oxygen, that aggregates in mitochondria. Maneb Mitochondrial complex III is inhibited.	^43,44^
Genetic Factors	SNCA anomalies in the anatomy of the mitochondria reduction in Complex I functioning Parkin Reduced ETC enzyme activity Numerous complexes I and IV subunits' protein levels drop. reduction in mitochondrial stability PINK1 Reduced ETC enzyme activity Reduced ATP synthesis Reduced mitochondrial fission architecture of the mitochondria is distorted DJ-1 Activities in Complex I and II decrease Decreases in ATP synthesis, oxygen uptake, and mitochondrial membrane potential mitochondrial morphological error Assembling error with complex I LRRK2 Reduced ATP synthesis and mitochondrial membrane potential fission/fusion dynamics errors	^45,46,47^
Oxidative imbalance	Destructs the substantia nigra protein peroxidation DNA oxidation.	^48^
Excitotoxicity	inadequate mitochondrial calcium storage insufficient calcium ATPase action prolongation of calcium transients. Minimized dopaminergic transmission from the nigrostriatal pathway Hyperactivity of the subthalamic nucleus	^49^
Apoptosis	DNA segregation apoptotic chromatin changes in dopaminergic neurons Increased growth of caspase-3	^50^

## Therapeutic advances for Neurodegenerative Disorders associated with mitochondria

 Mitochondrial malfunction and oxidative damage are involved in aging and neurodegenerative disorders, based on plenty of investigations. Because damaged mitochondria worsen and contribute to the development of neurodegenerative illnesses, it would seem sensible to use mitochondria-targeted medication as a therapy to cure or postpone the beginning of these diseases’ pathophysiology pathways. Agents that target oxidants and enhance mitochondrial ATP, reduce the formation of free radicals and oxidative damage, and promote better physiology of the mitochondria can be used to heal these mitochondrial pathological processes, which improve synaptic branching in neurons. As noted in the article, numerous mitochondria-targeted treatments such as Mitochondrial transplantation, mitochondrial gene therapy, redox therapy, etc. have been explored for their potency and efficacy.

 Several organizations have recently used antioxidant-rich meals to cure mice models of neurological conditions, which include vitamin C, E, coenzyme Q10 (CoQ10), and other herbal preparations.^51^ As a possible medicinal target, mitochondria are now being targeted by new therapeutic techniques.

###  Mitochondria transplantation

 Energy metabolism deficiencies, oxidative damage, calcium equilibrium instability, neuronal damage, and even death can all occur from mitochondrial failure. As a result, medicines that can maintain mitochondrial anatomy and physiology are thought to be effective in decreasing the disease’s course, particularly PD.^52^ However, because mitochondrial proteins and DNA sequences are irreparably damaged or altered in the course of neuronal illness,^53^ the medicines can only provide minimal neurological safety. Since mitochondria might trigger impulses for cell viability, a potentially promising therapy involves transferring mitochondria into the impaired brain region.^54^ Mitochondrial transplantation is gaining traction as a potential therapy for preserving mitochondrial activity following injury and thereby optimizing long-term functional status.^55^ Zhang and colleagues^56^ have transplanted muscle-derived homologous mitochondria into the lateral ventricles of mice’s brains. They discovered that transplanting mitochondria into the ventricular system minimized cell oxidative stress and death, reduced brain infarction extent, corrected neurological impairments, inhibited reacting astrogliosis, and enhanced neurogenesis. After an ischemia episode, mitochondria were recently found to transport between astrocytes and neurons,^57^ implying that astrocytes can discharge mitochondrial particles in the extracellular environment and invade neurons to maintain cell survival and recovery. Furthermore, the investigators discovered that the transplanted mitochondria’s mtDNA increased genes involved in angiogenesis and blood-brain barrier function, implying that the transplanted mitochondria can restore endothelial cells’ functioning.^58^ Improvements in ATP manufacture and calcium balancing capability; reductions in total ROS formation; and generation of a new pool of mtDNA are some of the processes through which transplanted mitochondria exhibit favorable effects.^59^

 The idea of repairing lost and dysfunctional mitochondria with good health external mitochondria has already been tested, and the outcomes have been convincing. The concern of an immune reaction, which would be a worry following mitochondrial implantation, is an essential consideration. The development of a technique for delivering drugs for transferring mitochondria to targeted regions and into target tissue is critical for furthering the development of traditional mitochondrial transplantation.^60^

###  Transplantation techniques

 External mitochondria have indeed been successfully transplanted into diverse recipient cells in diverse settings, and several reports have highlighted that exogenous mitochondria may be incorporated both in vitro and in vivo via co-incubation, direct injection, and cell-mediated transmission.

####  Sources and techniques for isolating mitochondria

 A suitable option for mitochondrial transplantation has been indicated as isolated mitochondria from skeletal muscles. The bestplaces to get mitochondria are the pectoral muscle, abdominal muscle, gluteal muscles, and sometimes even neck strap musculature like the sternohyoid, deltoid, and latissimus dorsi.^61^ Pectoral muscles are not a good site for women to extract mitochondria owing to cosmetic reasons.^62^ Recently, the idea has circulated that stem cells, particularly mesenchymal stem cells (MSCs), are a desirable and effective alternative source for isolating mitochondria.^63^ Intriguingly, it has been demonstrated that the secretome, which contains a variety of chemicals, especially mitochondria, can mediate the medicinal value of MSCs.^64^ Additionally, MSCs use tunnelling nanotubes to give their mitochondria to damaged cell.^65^ In individuals with mitochondrial disorders in particular, stem cell-derived mitochondria implantation may be a unique treatment option for tissue damage.^66^

###  Transplantation mechanism

####  In vitro

 Even though earlier research employed injections with a point diameter of 1 µm or less, straight microinjection of mitochondria into cell cultures was deemed impracticable considering the size of mitochondria relative to the diameter of injecting syringes. To address this issue, mitochondria were first inserted into oocytes to facilitate the use of larger dimension needles; after that, a mitocytoplast encompassing the cellular membranes, cytoplasm, and already administered mitochondria was removed from the oocyte. The recipient mitocytoplast can then be combined with this mitocytoplast.^67^ The concept of mitochondrial translocation by coincubation has also been examined, along with the direct transmission procedures into host tissue. When extracted mitochondria is co-incubated with cells in vitro, the xenogenic transmission of mitochondria was seen, leading to the effective insertion of exogenous mitochondria.^68^

 Co-culturing lung epithelium cells with defected and/or exhausted mitochondria with normal living beings bone marrow stem cell findings in donors of stable stem cell mitochondria with properly functioning mitochondria, as evidenced by higher ATP synthesis and the transformation of mitochondrial protein COXII from one cell to another transmission frameworks.^69^ It’s worth noting that effective mitochondrial transference through one cell to the other seems to be more common whenever the transference is from an active cell to the malfunctioning and/or deficient mitochondria.

####  In vivo

*In vivo* studies of phylogenetic maps and mitochondrial transplanting have also been carried out on many animal species models. A study that sequenced the mitochondrial genomes of several canine breeds with canine transmittable venereal tumor (CTVT) found an unusually high variation frequency in the CTVT specimens, which was considered to be induced by mitochondria translocation via canines in the malignant cells. Researchers hypothesis says host mitochondria are stronger than CTVT mitochondria, resulting in mitochondrial transference and an improvement in CTVT metabolic health. This notion is based on an endogenous process of mitochondrial transmission from one cell to cell *in vivo. In vivo* mitochondrial transfer from one cell to another has now been noticed in addition to the immediate injections. The researchers prompted astrocytes to discharge mitochondria in vitro by overexpressing the CD38 protein and discovered that adding these astrocyte-generated mitochondria to a template of nerve cellular damage in vitro increased nerve cell ATP output and cell viability, as well as those mitochondria, that were integrated into the neurons.^70^

###  Mitochondrial transplantation mechanism

 Extracellular vesicles (EVs), tunneling nanotubes (TNTs), and cellular fusion are all used to transport mitochondria between cells. In the bloodstream and prepared cell culturing environments, cellular and cytoplasmic membrane-free respiratory capable mitochondria have been discovered recently.^71^

####  TNT-mediated mitochondrial transfer

**Figure 6:**
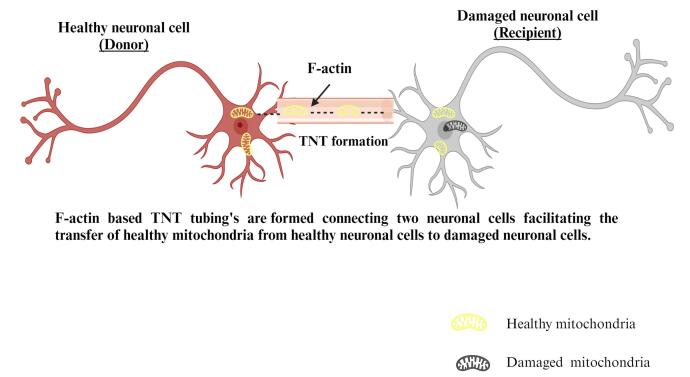
Mechanism of TNT-mediated mitochondrial transfer

 TNTs 12 are interstitial, actin, or microtubule-based intracellular tubes encased in a periplasm that interconnect cells and form junctional transportation systems (Figure 6). Actin-based TNTs (AC-TNTs) is created by thin filaments of F-actin, while MT-TNTs are formed by a thicker subset (0.7 mm) of both F-actin and microtubules carrying TNTs.^72^ AC-TNTs have a limited life and only transport tiny molecules, structures, and ions, while MT-TNTs have a bigger width, a longer life, and transport bigger organelles like mitochondria. Miro1 is important for mitochondrial transportation, according to molecular pathways of mitochondrial transfer. Miro1 deficiency slowed mitochondrial transport across TNTs and rendered the MSC’stherapeutic efficacy ineffective. Connexin 43 modulates TNT creation, according to,^73^ because knocking it out reduced TNT production in humans induced pluripotent stem cells and generated MSCs. Following tubulin or endocytosis suppression, mitochondrial transport was diminished in all co-cultures.^74^

####  EVs mediated mitochondrial transfer

**Figure 7:**
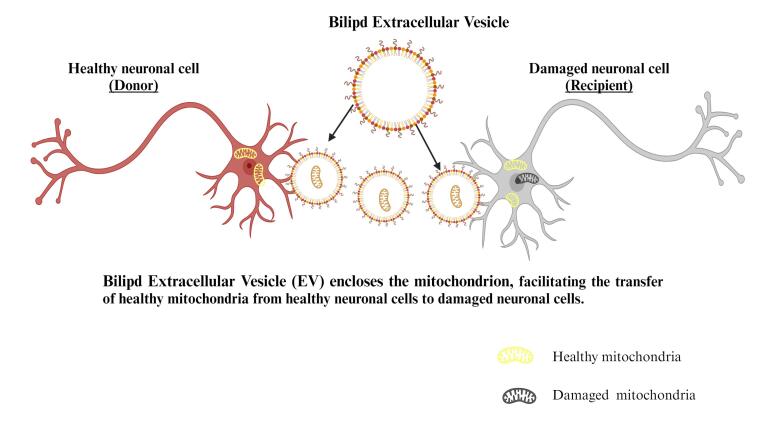
Mechanism of EVs mediated mitochondrial transfer

 EVs are a diverse set of bilipid cellular membranes that encase peptides, genetic information, and organelles such as mitochondria, ribosomes, and proteasomes.^75^ To facilitate intercellular cross-talk, EVs transport molecules and components to recipient cells. Massive EVs (microparticles) with a diameter of 100–1,000 nm can enclose mitochondria with a diameter of approximately 500 nm.^76^ The authors demonstrated that EVs were involved in the translocation of mitochondria between MSCs and macrophages (Figure 7).^77^ Extracellular aggregates, including mitochondria, were also found in prepared media from rat cortical astrocytes in vitro. In an animal model of cerebral ischemia, the researchers also revealed that astrocytes can discharge functioning mitochondria into neurons.^78^ Enclosing mitochondria into EVs, according to Morrison et al^79^ might be a safeguarding method against oxidative stress and the elimination of depolarized mitochondria.^80^

####  Cell fusion mediated mitochondrial transfer

**Figure 8:**
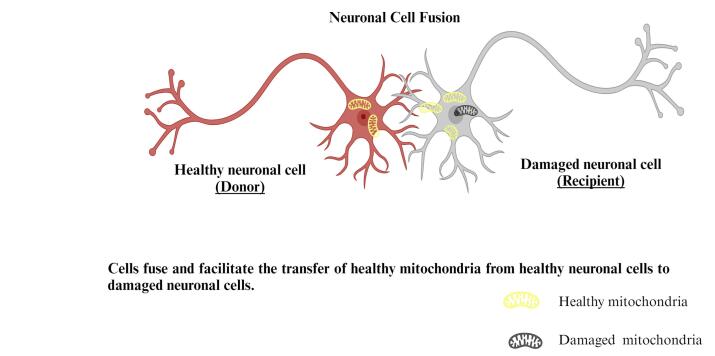
Mechanism of cell fusion mediated mitochondrial transfer

 Multiple investigations have shown that stem cells could merge with neuronal cells,^81^ resulting in hybrid cells that replicate stem and distinct cell characteristics.^82^ The extent of the intercultural link was found to be inversely proportional to the concentration of mitochondria transported: as the range across cells grew longer, fewer mitochondria were transferred. Large mitochondrial transport into targeted cells occurs as a consequence of cell fusion (Figure 8).^83^

###  Mitochondrial transplantation therapeutic applications in PD

 Emerging research shows that mitochondria might well be released from astrocytes and penetrate neurons to assist cell growth and regeneration.^84^ Muscular autologous mitochondrial implantation has been carried out by Zhang and colleagues^56^ in the lateral ventricles of mouse brains. They replaced degraded mitochondria following an ischemic stroke with transplanted mitochondria using a model of the middle cerebral artery (MCA)occlusion. They demonstrated that mitochondrial transplantation into the ventricular system minimized cellular oxidative load and death, lowered the size of infarcts in the brain, corrected cognitive impairments, slowed responsive astrogliosis, and stimulated neurogenesis. They discovered that superior neurological results were connected with higher levels of functional mitochondria in the cerebrospinal fluid. It has been noted that after transplant, energy output, mitochondrial protein translation, and enzyme activity enzymes are all enhanced. In cells saved by cell-to-cell mitochondrial pass on, one study discovered a three-fold rise in ATP concentration and a decline in lactate levels, showing that the human cells with reduced mtDNA were capable of transitioning from anaerobic to aerobic cellular respiration upon obtaining mitochondria.^85^

 Another research demonstrates that intravenously administered mitochondria can stop the advancement of PD by enhancing the levels of the ETC, lowering the threshold of oxidative stress, and limiting cellular injury.^86^ This research has used an MPTP generated PD rat model. The results lend support to the treatment approach of an i.v. or i.m. mitochondrial supplement. The origin and integrity of the isolated mitochondria, the administration method, and also the cellular uptake of additional mitochondria to enable effective neural ingestion inside the brain, are likely elements of an effective mitochondrial transplant.^87^

## Other therapeutic strategies involving mitochondria

###  Mitoquinone ( MitoQ )

 Co-enzyme Q10 and a triphenylphosphonium (TPP) cation make up the mitochondria-targeted anti-oxidant mitoquinone (MitoQ), which is more effective than untargeted anti-oxidants at mitigating mitochondrial oxidative injury due to its ability to aggregate several hundred-fold inside the mitochondria.^88^ The ability to permeate biological membranes and accumulate in large concentrations in mitochondria is facilitated by the lipophilic feature, which has an impact that is countless times greater than that of regular antioxidants.^89^ New research suggests MitoQ has positive impacts on disorders brought on by redox imbalances linked to neurodegeneration. According to Kang et al, MitoQ prevented intervertebral deterioration by correcting the mismatch in mitochondrial dynamics and enhancing PINK-mediated mitophagy.^90^ In Parkinson’s animal studies, MitoQ reduced the amount of caspase-3 activation that was brought on by MPP + and reduced the amount of mitochondrial aconitase deactivation that was caused by MPTP, keeping the TCA functioning normally and reducing oxidative injury.^91^ In one study, high doses of MitoQ were administered to PD animal models for up to 28 weeks, and the results indicated no evidence of damage to whole-body biology. This finding suggested that MitoQ, an antioxidant that targets the mitochondria, can be given to mice over an extended period without harm.^92^

###  Mitochondrial gene therapy

 Due to the high prevalence of problematic mtDNA in neurodegeneration, research aimed at alleviating mtDNA lesions, particularly genomic modification of mtDNA and its downstream genes, is required. It has been introduced that intervening in the neurodisorder patient’s genetic makeup might be a powerful tool for effectively correcting photogenic processes, mildening and alleviating clinical signs, repairing degraded neurons in the brain circuits, and preventing neuronal death.^93^ There’s been a range of possible advancements in this research, which can be grouped into three distinct groups: targeted suppression of defective mtDNA, recombinant mtDNA replacement, and allotropic production of mitochondrial proteins. Antisense suppression of genetically mutant mtDNA and restriction endonuclease selection for mtDNA eradication are two well-studied techniques for selective suppression of mutant mtDNA. Antisense inhibition works by genetically blocking aberrant mtDNA replication and thereby reducing the production of faulty proteins.^94^

 Another method for mitochondrial genome editing is to replace faulty mtDNA with recombinant mtDNA utilizing gene-carrying carriers. Not just heteroplasmy but also homoplasmy will profit from the substitution of faulty mtDNA with “good” recombinant mtDNA gene. Recent studies on the use of allotropic regulation of mitochondrial proteins show that this strategy has a bright future. An investigation reveals that introducing NDI1 into mitochondria recovers NADH dehydrogenase function and, as a consequence, prevents neuronal destruction in the substantia nigra of PD rat models.^95^

###  Redox therapy

 Oxidative stress has been extensively explored as a therapy method for neurodegeneration, built on the notion that it is among the most common mitochondrial disorders associated with neurodegenerative disorders, as well as the ease with which an antioxidant strategy can be implemented. Glutathione, CoQ10, vitamin E, and C are minor reducing chemicals that play a significant part in mitochondria’s natural defense plan against oxidants. In Parkinson’s, the expression of these molecules is reduced. Various research teams have expressed interest in the augmenting these tiny reducing agents to detoxify oxidative stresses in neurodegenerative disorders.^96^

*Coenzyme Q:* Also called CoQ10, ubiquinone, and ubidecarenone, is found mostly in the interior layer of mitochondria. Ubiquinone, a lipid-soluble benzoquinone with antioxidant activities once reduced to ubiquinol or by CoQ10, promotes rise in vit E. It is placed in the inner membrane of the mitochondria, is required for electron transfer events in Complex I and II throughout oxidative phosphorylation, and has a significant impact on the formation of ATP.^97^ In afflicted areas of preclinical models of PD, seizures, and ischemia, CoQ10 protects to neuronal cells from oxidative stress CoQ10 has been extensively studied as a treatment for neurological illnesses, both in preclinical studies and clinical trials, investigations due to its multiple mitochondrial therapeutic properties and severe mitochondrial dysfunction.^98^

*Vitamins C and E:* Vitamin C is a potent cytoplasmic reducing agent, and vitamin E is a key absorber of lipid oxidation in the brain; both are necessary vitamins for normal physiological functions. *In vivo* and in vitro, ascorbic acid and alpha-tocopherol have been demonstrated to safeguard neurons from the consequences of oxidative overload.^99^ Besides that, replenishment with vitamin E alone or in conjunction with vitamin C and vitamin E can minimize the risk of PD, while the integration of vitamin E and vitamin C has been confirmed to decrease the occurrence of PD in the elderly population.^100^ The pairing of vitamins E and C have been proven to slow the growth of PD.^101^ When analyzing the inconsistencies in vitamin treatment outcomes, it is suggested that the restrictions of vitamin therapy may have an impact on its efficacy, causing the variance. The drawbacks involve (1) poor transmission of vitamin E and vitamin C across the BBB, which makes it difficult to accumulate curative vitamin concentrations in neurons in the brain mitochondria; (2) quick combustion of vitamin C, which reduces its adequacy in treatment; and (3) possible risks of high Vitamin E intake. To circumvent these limitations, mitochondrial-targeted vitamin E is designed.^102^

*Physical fitness:* Several studies suggest that physical activity can help slow the progression of neurodegeneration, which might be linked to exercise-induced mitochondrial function recovery.^103^ Exercise increases mitochondrial processes in the brain. Exercise improves mRNA expression of TFAM and Ndufa6, components of mitochondrial complex I, as well as tolerance to rotenone, an antagonist of complex I performance. Simultaneously, exercise enhances mtDNA restoration potency in the rat hippocampus and stimulates mitochondrial uncoupling proteins, which can control mitochondrial proliferation and reduce mitochondrial-generated ROS production.^104^ Mitophagy is improved, which reduces the number of mitochondria that are faulty in cells and maintains high mitochondrial quality.

## Conclusion

 Regarding the mitochondria-targeted therapies stated above, modifications to mitochondrial dynamics and apoptosis forces retain potential as prospective beneficial therapeutic techniques, based on existing experiments conducted. Several therapeutics are now being examined on individuals with neurodegenerative diseases, and some have already proven to be effective in medical care. Therapeutic target methods are believed to assist mitochondria in being able to deal better with oxidative damage, excitotoxicity, and other neurological stressors, as well as ensure effective respiratory activity in neurons. Thorough testing is presently being carried out with the goal of better comprehendingcomprehend better the molecular process of mitochondrial participation in hereditary neurodegenerative illnesses, as well as to finding viable treatments using focused techniques and massive drug testing. Optimizing mitochondrial dynamics may help in the growth of novel therapeutic for mitochondria-directed treatments in PD. We anticipate that mitochondrial transplantation will become a valuable therapeutic option for healthcare practitioners treating disorders caused by mitochondrial abnormalities. The isolation methodology, quality of separated mitochondria, and tissue-specific variable uptake all play a role in the therapeutic result of mitochondrial transplantation. The proportion of functioning mitochondria following isolation, as well as the integrity of the mitochondria throughout time, are critical indicators of the effectiveness of neural regeneration. The function of changed mitochondrial transmembrane characteristics in brain aging and degeneration requires further investigation, which should include mitochondrial sub-membrane fractions.

## Acknowledgments

 We would like to express our sincere gratitude to the Department of Pharmacy, Pranveer Singh Institute of Technology, Kanpur, for providing the necessary facilities for compiling this review article.

## Competing Interests

 The authors declare no conflicts of interest, financial or otherwise.

## Ethical Approval

 Not applicable.

## Funding

 None.
